# Therapeutic Targeting of the Mitochondria Initiates Excessive Superoxide Production and Mitochondrial Depolarization Causing Decreased mtDNA Integrity

**DOI:** 10.1371/journal.pone.0168283

**Published:** 2016-12-28

**Authors:** Kaytee L. Pokrzywinski, Thomas G. Biel, Dmitry Kryndushkin, V. Ashutosh Rao

**Affiliations:** Laboratory of Applied Biochemistry, Division of Biotechnology Research and Review III, Office of Biotechnology Products, Center for Drug Evaluation and Research, U.S. Food and Drug Administration, Silver Spring, Maryland, United States of America; University of PECS Medical School, HUNGARY

## Abstract

Mitochondrial dysregulation is closely associated with excessive reactive oxygen species (ROS) production. Altered redox homeostasis has been implicated in the onset of several diseases including cancer. Mitochondrial DNA (mtDNA) and proteins are particularly sensitive to ROS as they are in close proximity to the respiratory chain (RC). Mitoquinone (MitoQ), a mitochondria-targeted redox agent, selectively damages breast cancer cells possibly through damage induced via enhanced ROS production. However, the effects of MitoQ and other triphenylphosphonium (TPP^+^) conjugated agents on cancer mitochondrial homeostasis remain unknown. The primary objective of this study was to determine the impact of mitochondria-targeted agent [(MTAs) conjugated to TPP^+^: mitoTEMPOL, mitoquinone and mitochromanol-acetate] on mitochondrial physiology and mtDNA integrity in breast (MDA-MB-231) and lung (H23) cancer cells. The integrity of the mtDNA was assessed by quantifying the degree of mtDNA fragmentation and copy number, as well as by measuring mitochondrial proteins essential to mtDNA stability and maintenance (TFAM, SSBP1, TWINKLE, POLG and POLRMT). Mitochondrial status was evaluated by measuring superoxide production, mitochondrial membrane depolarization, oxygen consumption, extracellular acidification and mRNA or protein levels of the RC complexes along with TCA cycle activity. In this study, we demonstrated that all investigated MTAs impair mitochondrial health and decrease mtDNA integrity in MDA-MB-231 and H23 cells. However, differences in the degree of mitochondrial damage and mtDNA degradation suggest unique properties among each MTA that may be cell line, dose and time dependent. Collectively, our study indicates the potential for TPP^+^ conjugated molecules to impair breast and lung cancer cells by targeting mitochondrial homeostasis.

## 1. Introduction

The unique physical properties of mitochondria in cancer cells substantiate the therapeutic potential for pharmacological agents that selectively accumulate in mitochondria as a targeted strategy to ameliorate the disease [1]. Cancer cell mitochondria have been classified as having elevated reactive oxygen species (ROS) levels [1, 2]. Although this trait is not exclusive to cancerous cells, it is a classic hallmark of an energetic imbalance at the cellular level which is a common signature of different pathological concerns including cancer, aging, and neurodegenerative disease [2]. While elevated basal ROS levels in cancer cells do not induce cell death, excessive ROS can lead to the unintended oxidation of nucleic acids, proteins and lipids that in turn could alter metabolic functions in rapidly dividing cancer cells [1]. For these reasons, compounds that selectively accumulate in the mitochondria and alter redox homeostasis are appealing as chemotherapeutics. However, information on the mechanism(s) of how mitochondria-targeted redox-active agents influence mitochondrial homeostasis is currently lacking.

Reactive oxygen species (ROS) are natural byproducts of mitochondrial oxidative phosphorylation (OxPhos). Uncoupling oxidation from phosphorylation, in a variety of ways, can lead to the leakage of electrons from complex I, II or III, which in turn can prematurely reduce oxygen and result in the formation of superoxide [3–6]. Dysregulation of the respiratory chain is known to induce excess mitochondrial ROS which can ultimately lead to the damage and degradation of macromolecules essential to mitochondrial function. Mitochondrial DNA (mtDNA) and proteins are particularly sensitive to ROS as they are located in close proximity to the respiratory chain. mtDNA is also more susceptible to oxidative damage than nuclear DNA (nDNA) as it lacks histones which are known to provide protection from ROS [7, 8]. Additionally, mitochondria have limited DNA repair mechanisms making damage to mtDNA potentially more detrimental to mitochondrial physiology [9].

Oxidant-induced mtDNA damage and mutagenesis is of particular interest since it has been established as an underlying mechanism in cancer initiation and progression [10]. Oxidant-induced DNA damage is known to cause G to T transversions during replication and thereby propagate mutagenesis (discussed in [10]). The damage inflicted by ROS on mtDNA constitutes the free radical theory of aging [11, 12]. This theory has established that elevated mitochondrial ROS levels lead to increased mtDNA damage and mutagenesis which in turn potentiate progressive respiratory chain dysregulation and ROS production thereby completing a ‘vicious cycle’ that ultimately leads to cell death. Additionally, it has recently been demonstrated that oxidant-induced mtDNA damage can result in the loss of mtDNA likely due to the fact that mitochondria contain multiple copies of the mtDNA genome and possess limited DNA repair mechanisms [10].

Although the majority of mitochondrial genes are encoded in the nucleus, a total of 37 genes are transcribed by the ~16 kb mitochondrial genome including 13 proteins, 22 tRNAs and 2 rRNAs. The mitochondrial-encoded rRNAs and tRNAs are used in mitochondrial protein production both of which are critical for translation of the 13 mitochondrial proteins which encode essential subunits of respiratory chain complexes (RCCs) required for ATP production via OxPhos. Additionally, many (~70%) proteins essential to mitochondrial function are also encoded in the nucleus and are transported to the mitochondria through a charge dependent mitochondrial targeting sequence (MTS) (discussed in [13]). Therefore, mtDNA integrity, mitochondrial membrane potential (MMP) and reactive oxygen species production are directly linked to mitochondrial health and function.

Emerging research in cancer therapy is focused on exploiting the biochemical differences between cancer and non-cancer cell metabolism. Cancer cell mitochondria are known to have higher mitochondrial transmembrane potential as compared to normal cells [14]; therefore, there is a hypothetical rationale for targeting mitochondria with lipophilic, delocalized cation drugs to selectively deplete ATP levels in tumor cells [15, 16]. One such lipophilic cation is triphenylphosphonium (TPP^+^). TPP^+^ has been conjugated to naturally occurring compounds that can act as antioxidants such as co-enzyme Q [Mitoquinone (MitoQ)], vitamin E analogs [Mitochromanol-acetate (MitoCA)] or an SOD mimetic [MitoTEMPOL (MitoT)] among others [16–18]. These compounds were originally designed to boost local antioxidant function and thereby decrease mitochondrial oxidative damage [19, 20]. These compounds are being studied across multiple oxidant-induced disorders including cancer, neurodegenerative disorders, and aging among others [21]. MitoQ is among the first mitochondrial-targeted agent (MTA) to have undergone human studies, for Parkinson disease and for liver damage associated with hepatitis C [22–24].

Several antioxidants including sulphoraphane, ascorbic acid (reviewed in [25]) and MitoQ [17, 26] among others, have been shown to exhibit pro-oxidant properties *in vitro* and have variable effects on cancer cell survival and disease progression. In order to understand the therapeutic potential of selective mitochondrial antioxidants (which have the potential to function as pro-oxidants), we conducted a detailed investigation of the effects of three TPP^+^ conjugated redox-active therapeutics [16–18]: MitoT, MitoQ and MitoCA, on mitochondrial homeostasis in triple negative breast [(MDA-MB-231) TNBC] and small cell lung [(H23) SCLC] carcinoma cell lines. All of these compounds are known to be cytotoxic to breast (MDA-MB-231) and lung (H23) cancer cell lines at concentrations exceeding 5μM (S1 and S2 Figs [15–17]). Additionally, studies on MitoQ and MitoCA have shown no effect on non-cancerous mammary epithelial cell lines including MCF-12A (MitoQ) and -10A (MitoCA) at concentrations below 10μM [16, 17]. These studies have yet to be performed on MitoT.

In the present investigation, we utilized sub-toxic concentrations of MTAs (2μM) and investigated their early effects on mitochondrial health by measuring mitochondrial superoxide production and MMP. We then investigated mtDNA integrity by examining mtDNA damage via fragmentation and copy number assays, and measuring transcript and protein levels of proteins known to be essential for the structural stability (SSBP1 and TFAM), transcription (POLRMT) and replication (TWINKLE and POLG) of mtDNA. Finally we looked for changes in mitochondrial metabolism by measuring aconitase TCA cycle activity along with transcript and protein levels of RCC subunits. Lastly, we evaluated mitochondrial bioenergetics by measuring oxygen consumption and extracellular acidification rates. The MTAs tested in this investigation caused the rapid induction of superoxide production and/or depolarization of the mitochondrial membrane, which likely drive the downstream effects observed on mtDNA integrity, mitochondrial metabolism and overall mitochondrial homeostasis. Collectively, our findings demonstrate that these TPP^+^ conjugated molecules, although initially designed as antioxidants, exhibit pro-oxidant activity and therefore may have a potential role as therapeutics for specific types of TNBC and SCLC.

## 2. Materials and Methods

### 2.1 Chemicals

MitoTEMPOL (MitoT), mitoquinone (MitoQ), and mitochromanol-acetate (MitoCA) were kindly provided by Drs. Joy Joseph and Balaraman Kalyanaraman at the Medical College of Wisconsin (Milwaukee, WI), and their synthesis was described previously [16–18]. All MTAs possess a TPP^+^ moiety for targeting the mitochondria, a carbon linker for penetrating the lipid membranes and a redox reactive moiety for catalyzing redox reactions. MitoT functions as a superoxide dismutase mimetic, MitoQ as a ubiquinone derivative and MitoCA as a vitamin E analog [16], all are conjugated by four (MitoT) or ten (MitoQ and MitoCA) carbon chain linkers to TPP^+^ [16–18]. The following chemicals were used as positive or negative controls: TPP^+^, Antimycin A (Am-A), Carbonyl cyanide 3-chlorophenylhydrazone (CCCP) and Oligomycin (Oligo) (130079, A8674, C2759 and 75351, respectively; Sigma Aldrich; St. Louis, MO).

### 2.2 Cell Cultures and Experimental Design

The TNBC cell line MDA-MB-231 and the SCLC cell line H23 were obtained from the American Type Culture Collection (ATCC; Manassas, VA). All experiments were conducted in triplicate for 1 to 24 hours with final concentrations (*v*/*v*) of 0.02% DMSO or 2μM of MitoT, MitoQ or MitoCA along with the appropriate positive controls when necessary. All experiments were conducted on cultures that were 80–90% confluent.

### 2.3 Mitochondrial Superoxide Production

Mitochondria-targeted MitoSOX™ Red fluorogenic dye (M36008; Thermo Fisher Scientific; Rockford, IL) was used to measure mitochondrial superoxide accumulation according to the manufacturer’s instructions. Antimycin-A (40μM) was used as a positive control. MitoSOX fluorescence was measured at 510nm excitation and 580nm emission on a SpectraMax® i3 microplate reader (Molecular Devices; Sunnyvale, CA). MitoSOX fluorescence was adjusted based on cell number as determined by the sulforhodamine B assay (as described in [27]) measured at an absorbance of 565nm.

### 2.4 Mitochondrial Membrane Potential

MMP was measured using the JC-1 assay (T3168; Thermo Fisher Scientific) according to manufacturer’s protocol as described previously [17] in 96-well plates on a SpectraMax i3® microplate reader with an excitation of 485nm and emission at 540nm and 590nm. CCCP (50μM) was used as a positive control. MMP was determined using the ratio of the fluorescence of J-aggregates (590nm) to monomers (540nm) and normalized to the respective DMSO control.

MDA-MB-231 cells were seeded on glass bottom dishes (Cellvis; Mountain View, CA) and incubated with 300nM tetramethylrhodamine methyl ester (TMRM) (T5428; Sigma Aldrich) to label polarized mitochondria. Images were taken on an inverted Zeiss LSM 700 confocal microscope (Carl Zeiss; Oberkochen, Germany).

### 2.5 Mitochondrial DNA Damage and Copy Number

DNA was extracted with the FlexiGene DNA kit (51204; Qiagen) according to the manufacturer’s protocol. The DNA concentration and purity were determined on a Nanodrop2000c (Thermo Fisher Scientific).

Long-range polymerase chain reaction (PCR) was used to evaluate mtDNA damage [as described in ([28, 29]). A long (~10kb) and short (~117bp) DNA fragment were amplified using 16 cycle PCR with Long Amp Taq DNA Polymerase (M0323L; New England Biolabs; Ipswich, MA) or Taq DNA Polymerase (M0267L; New England Biolabs) [29]. PCR products were assessed using agarose gel electrophoresis (long) or PAGE (short). Band intensities were determined using densitometry and the damage index was calculated by the ratio of the long to short PCR-product band intensity, normalized to the DMSO treatment. Primer sequences can be found in S1 Table.

Mitochondrial copy number was assessed using the ratio of mitochondrial to nuclear (nDNA) DNA by amplification of short regions of β2-microglobulin [86bp (nDNA)] and tRNA_Leu_ [107bp (mtDNA)] (described in [30]). SYBR green qPCR was performed on a QuantStudio 6 Flex (Thermo Fisher Scientific) using the conditions recommended by the manufacturer. Primer sequences can be found in S1 Table.

### 2.6 Gene Expression Changes

To examine transcriptional changes in gene expression, RNA was extracted with the miRNeasy Micro kit (217004; Qiagen; Valencia, CA) according to manufacturer’s protocol with slight modifications as follows. Cells were suspended in QIAzol lysis reagent and loaded onto a QIAshredder spin column (79654; Qiagen) for consistent homogenization after which the RNA was extracted as recommended and the concentration/purity was determined on a Nanodrop2000c (Thermo Fisher Scientific).

Transcriptional changes were determined with real-time quantitative polymerase chain reaction (qPCR) using TaqMan assays (S1 Table). RNA was reverse transcribed using the High Capacity cDNA reverse transcription kit (4368813, Thermo Fisher Scientific). qPCR was performed on a QuantStudio 6 Flex (Thermo Fisher Scientific) using the conditions recommended by the manufacturer.

### 2.7 Protein (Total and Mitochondrial) Extraction

For total protein extraction, cells were lysed in RIPA buffer (89901; Thermo Fisher Scientific) containing protease inhibitors (11873580001; Roche Diagnostics; Mannheim, Germany) as recommended by the manufacturer. Protein concentration was determined via the bicinchoninic acid (BCA) assay (23225; Thermo Fisher Scientific).

Mitochondrial and cytoplasmic protein was extracted as described previously [31, 32] with slight modifications. Briefly, cells were re-suspended in hypotonic buffer and homogenized followed by two, five-minute centrifugations at 1,000 x g to remove whole cells. Sucrose was added to the supernatant as a membrane preservative. After high-speed centrifugation, the supernatant was retained as the cytosolic fraction. The mitochondrial pellet was washed and then lysed in RIPA buffer as in the total protein extraction method. Protein concentrations were determined by the BCA assay.

### 2.8 Immunoblot Analysis

Immunoblotting was performed as previously described [17] with slight modifications. Briefly, proteins were separated using SDS-PAGE and transferred to nitrocellulose membranes using a TransBlot^®^ Turbo^TM^ blotting system (Bio-Rad). Immunoblots were imaged using an Odyssey Infrared Imager (LI-COR). Antibodies can be found in S1 Table. Band intensities were calculated using densitometry using Image Studio Lite v4.0 (LI-COR).

### 2.9 Relative Aconitase Activity

Mitochondrial extracts (as in *Section 2*.*7* before RIPA lysis) were assayed for aconitase activity using the Aconitase Enzyme Activity Assay kit (ab109712; Abcam; Cambridge, MA) according to manufacturer’s instructions. The assay measures aconitase activity through the conversion of isocitrate to cis-aconitate. Aconitase activity was determined by the change in absorbance over time after normalization to total mitochondrial protein, and normalized to the DMSO control.

### 2.10 Mitochondrial Oxygen Consumption Rate

Mitochondrial oxidative respiration was evaluated by monitoring the oxygen consumption rate (OCR) and the extracellular acidification rate (ECAR). Oligomycin (1μM) was used as a positive control. OCR/ECAR was measured for 40 minutes on the Seahorse XF Analyzer according to manufacturer’s recommendations (Seahorse Bioscience; Billerica, MA).

### 2.11 Statistical Analyses

When appropriate, statistical analyses were generated with the program GraphPad Prism v6.05 (GraphPad; La Jolla, CA). A two-way ANOVA was used to determine differences in superoxide production, MMP and aconitase activity with time and drug treatment as factors. A Dunnett’s test for multiple comparisons was used to evaluate specific differences in drug treatment over time compared to DMSO treatment. A two-way ANOVA was also performed to determine differences in transcriptional changes with gene expression and drug treatment as factors. A Dunnett’s multiple comparison test was then used to evaluate differences in specific gene expression by treatment. Expression levels were considered biologically relevant if there was a significant p-value and a log2 fold change in expression exceeding 0.30 or a 23%. A one-way ANOVA was conducted to determine changes between drug treatments and the DMSO control for mtDNA damage, mtDNA copy number and OCR/ECAR experiments. Multiple comparisons were calculated using a Tukey’s Honestly Significant Difference post-hoc test for specific differences in responses to MTAs.

## 3. Results

### 3.1 Excessive Mitochondrial Superoxide Production

**Fig 1:**
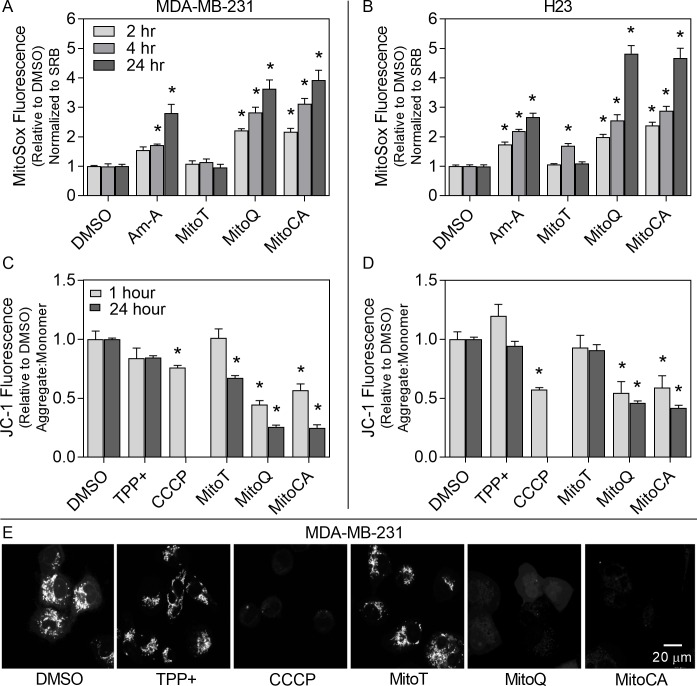
Mitochondrial superoxide production and MMP. MDA-MB-231 (A,C,E) and H23 (B,D) cell lines were exposed to either DMSO (0.02%), TPP^+^ MitoT, MitoQ or MitoCA at 2μM, Antimycin A (Am-A) at 40μM or CCCP at 50μM for 1 or 2 (light gray), 4 (gray) or 24 (dark gray) hours. In A-B, superoxide levels were assessed using MitoSOX normalized to total protein determined by the SRB assay. In C-D, MMP was assessed using the JC-1 assay by calculating the ratio of aggregates (590nm) to monomers (540nm). Bars represent the average normalized fluorescence per cell in at least two independent experiments (n = 4–10) relative to the DMSO control +/-1 SEM. Asterisks denote statistical significance at p<0.05 relative to the respective DMSO control at each time. TMRM imaging (E) was used to confirm changes in MMP in MDA-MB-231 cells after 1 hour drug exposure (as in C-D). Scale bar is 20μm.

We used MitoSOX to test whether TPP^+^-conjugated drugs that accumulate in the mitochondria lead to production of excessive mitochondrial superoxide (Fig 1A and 1B). The complex III inhibitor Antimycin A enhanced superoxide production in both cell lines (p<0.05). Consistent with our previous findings [17], MitoQ enhanced superoxide production by 2.2 to 3.6 fold in MDA-MB-231 cells between 2 and 24 hours (p<0.05; Fig 1A). MitoCA also enhanced superoxide production in MDA-MB-231 cells by 2.0 to 4.8 fold between 2 and 24 hours (p<0.05). However, MitoT treatment did not cause a change in superoxide levels. These data suggest that MitoQ and MitoCA rapidly induced mitochondrial superoxide production as early as 2 hours exposure in MDA-MB-231 cells.

Mitochondrial superoxide production was also evaluated in H23 cells after exposure to MTAs (Fig 1B). H23 cells displayed higher basal levels of superoxide than MDA-MB-231 cells which may account, in part for some of the cell type differences in this study (S3 Fig). MitoQ and MitoCA enhanced superoxide production between 2 and 24 hours by 2.0 to 4.8 fold (p<0.05). However, MitoT treatment had no effect on superoxide levels at 2 or 24 hours post treatment but did cause an increase in superoxide at 4 hours (p<0.05; Fig 1B). These data suggest that MitoQ and MitoCA may enhance mitochondrial superoxide production in both MDA-MB-231 breast and H23 lung cancer cells and that MitoT may have differential effects on superoxide production reliant on the cancer type.

### 3.2 Mitochondrial Membrane Potential

To investigate MTAs effects on mitochondrial function, we measured the MMP using JC-1 fluorometry (Fig 1C–1E). The mitochondria depolarizing agent CCCP resulted in a greater than 24% reduction in JC-1 aggregate formation at 1 hour in both cell types (p<0.05). Consistent with our previous findings [17], MDA-MB-231 cells treated with MitoQ for 1 or 24 hours induced a 56 to 74% loss in JC-1 aggregate formation (p<0.05; Fig 1A). Treatment with MitoCA caused a 43 to 75% loss in aggregate formation between 1 and 24 hours exposure (p<0.05). At 1 hour, MitoT treatment had no effect on MMP, but after 24 hours there was a 33% loss in aggregate formation (p<0.05). Changes in MMP due to MTAs were confirmed in MDA-MB-231 cells using TMRM imaging (S4 Fig and Fig 1E).

To expand these findings into a different cancer cell line, H23 cells were subjected to the same MTA treatments (Fig 1D). MitoQ treatment caused 45 to 54% reduction in JC-1 aggregates between 1 and 24 hours exposure (p<0.05). MitoCA caused a similar loss at 1 and 24 hours in aggregate formation with a 41% to 58% reduction (p<0.05). However, MitoT treatment had no effect on MMP (Fig 1D). Collectively, these data suggest that both MitoQ and MitoCA induced rapid (<1 hour) mitochondrial depolarization in the breast (MDA-MB-231) and lung (H23) cancer cell lines evaluated, but that MitoT induced mitochondrial depolarization was selective for the breast cancer cells and occurred at a later time point (24 hours).

### 3.3 mtDNA Damage and Copy Number

**Fig 2:**
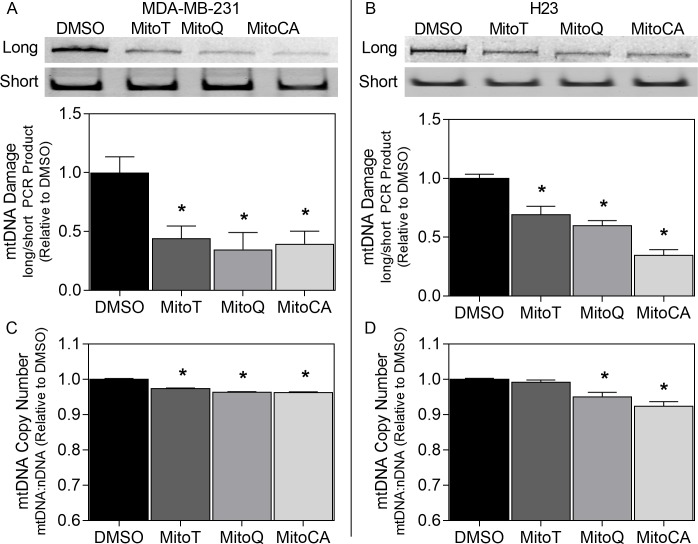
Mitochondrial DNA integrity. mtDNA damage (A-B) and copy number (C-D) in MDA-MB-231 (A,C) and H23 (B,D) cells after exposure to DMSO [(0.02%) black bars], MitoT (dark gray bars), MitoQ (gray bars) or MitoCA (light gray bars) at 2μM for 24 hours. mtDNA fragmentation (A-B) was evaluated using PCR amplification of a long mitochondrial sequence relative to a short mitochondrial sequence. Band intensities of PCR products were quantitated using densitometry. In A-B, gels are representative images for PCR products. In C & D, mitochondrial copy number was assessed by amplification of short regions of house-keeping genes in both nDNA and mtDNA. Bars depict the mean ratio of long to short band intensities (A-B) or the mean ratio of mtDNA:nDNA (C-D) relative to the DMSO treatment +/-1 SEM. Asterisks show statistical significance at p<0.05 relative to the DMSO control at each time. For each assay, two independent experiments were performed (n = 3).

We hypothesized that MTAs may damage and destabilize mtDNA as a consequence of the excessive superoxide production and mitochondrial membrane depolarization observed with these treatments. Therefore, we investigated the extent of mtDNA damage using PCR amplification of a long and short mtDNA region [29]. A reduction in amplification of the long fragment would indicate mtDNA damage as DNA lesions are known to slow down or impede the progression of DNA polymerase (discussed in [33]). At 24 hours, MDA-MB-231 cells treated with all MTAs displayed a significant reduction (p<0.05) in amplification of the long fragment by 56–65% (Fig 2A). In H23 cells, all MTAs also significantly reduced (p<0.05) amplification of the long region by 31–65% (Fig 2B). Together, these results indicate that these TPP^+^ conjugated antioxidant agents enhance mtDNA damage in the breast and lung cancer cell lines evaluated.

To gauge the extent of mtDNA damage on mtDNA integrity, we measured mitochondrial DNA copy number. Relative changes in mtDNA copy number can be estimated by taking the ratio of short regions of housekeeping genes in both mtDNA and nDNA. Changes in mtDNA copy number are closely associated with changes in mitochondrial proteins important to mtDNA replication including TFAM, TWINKLE and POLG (23–25) among others. In MDA-MB-231 cells, all MTAs significantly reduced mtDNA copy number by 3–4% (p<0.05; Fig 2C). In H23 cells only MitoQ and MitoCA induced significant decreases in mtDNA copy number by 5–8% (p<0.05; Fig 2D).

### 3.4 mtDNA Gene Expression

**Fig 3:**
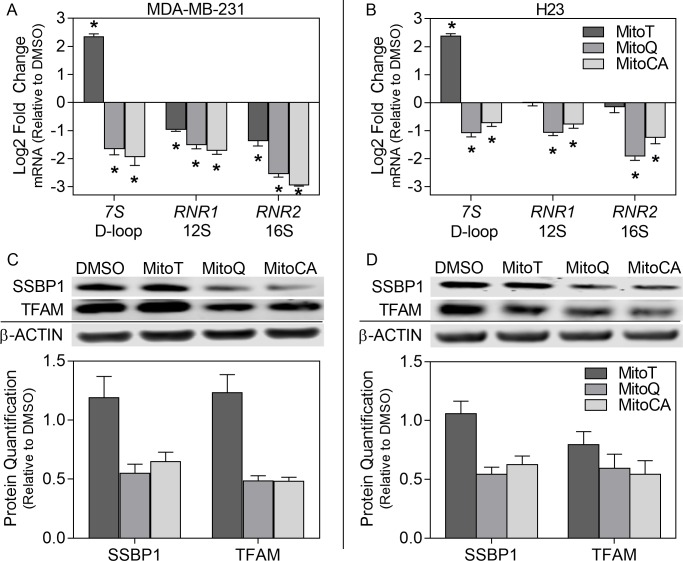
mtDNA gene expression and stability. Gene expression of *7S*, 12S (*RNR1*) and 16S (*RNR2*) transcripts (A-B) along with SSBP1 and TFAM protein levels (C-D) in MDA-MB-231 (A,C) and H23 (B,D) cell lines after 24 hours exposure to 0.02% DMSO or MitoT (dark gray bars), MitoQ (gray bars) or MitoCA (light gray bars) at 2μM. In A-B, bars represent the average log2 fold change normalized to *GAPDH* and the DMSO control. Statistical significance is expressed as asterisks at p<0.05 relative to the DMSO control. In C-D, bars signify the mean densitometry normalized to the corresponding DMSO treatment. For each endpoint, two independent experiments were performed (n = 3). Error bars signify +/-1 SEM.

To evaluate the downstream (24 hours) effects of mtDNA damage by TPP^+^ conjugated MTAs on mitochondrial physiology, we evaluated transcriptional changes in mitochondrial-encoded genes required for mtDNA displacement loop (D-loop) stability (*7S*) and mitochondrial ribosomal subunits (*RNR1* for 12S and *RNR2* for 16S). Comparative Ct qPCRs revealed a decrease in mRNA levels in MitoQ and MitoCA treated MDA-MB-231 cells for all transcripts [*7S*, *RNR* (12S) and *RNR2* (16S)] with log2 fold changes between -1.5 and -3.0 (p<0.05; Fig 3A). Treatment with MitoT resulted in a greater than 2.0 (log2) fold change increase in expression in *7S* but a concordant decrease in 12S (-0.98) and 16S (-1.4) transcripts, essential to ribosome formation (p<0.05; Fig 3A).

The effects of MTAs on mitochondrial physiology were evaluated in a second cancer cell line. H23 cells displayed a reduction in the mRNA levels of *7S*, 12S and 16S transcripts with log2 fold changes ranging between -1.1 and -1.9 for MitoQ and between -0.73 and -1.3 for MitoCA treated cells (Fig 3B). However, exposure to MitoT resulted in greater than 2.0 (log2) fold change increases in expression in 7S transcripts similar to the MDA-MB-231 cells (Fig 3A and 3B). Contradictory to MDA-MB-231 cells, MitoT treatment had no effect on either 12S or 16S transcript levels in H23 cells. Collectively, these data demonstrate that MitoQ and MitoCA induce changes in ribosomal subunit mRNA levels in both breast and lung cancer cells but also that MitoT explicitly impacts ribosomal subunit mRNA levels in breast cancer cells. Additionally, all MTAs have the potential to impact the stability of the D-loop in mtDNA nucleoids through changes in *7S* transcript levels in both breast and lung cancer cells, where MitoQ and MitoCA may destabilize and MitoT may preferentially stabilize the D-loop.

### 3.5 mtDNA Maintenance and Stability

To directly test the effect of MTAs on mtDNA maintenance, we investigated changes in the mitochondria-targeted, nuclear-encoded proteins essential to mtDNA stability including single stranded binding protein (SSBP1) and transcription factor A (TFAM). SSBP1 is important in maintaining the D-loop for mtDNA replication and transcription [34, 35]. TFAM is the key activator of mitochondrial transcription and also functions in mtDNA maintenance and mitochondrial homeostasis [36]. MDA-MB-231 cells treated with MitoQ and MitoCA resulted in decreases in SSBP1 by 35–45% and in TFAM by 51% (Fig 3C). Treatment of MDA-MB-231 cells with MitoT displayed the opposite effect and enhanced SSBP1 (19%) and TFAM (24%) protein levels (Fig 3C).

The levels of SSBP1 and TFAM were also evaluated in H23 cells after exposure to MTAs. MitoQ and MitoCA treatment caused reductions in SSBP1 by 37–46% and in TFAM by 41–46% (Fig 3D). H23 cells showed either no change (<10%) or a 21% reduction in SSBP1 and TFAM after treatment with MitoT, accordingly (Fig 3D). Collectively, these results support the activity of TPP^+^ conjugated MTAs towards abrogating mtDNA replication, transcription and overall stability in both breast and lung carcinomas.

### 3.6 mtDNA Replication Machinery

**Fig 4:**
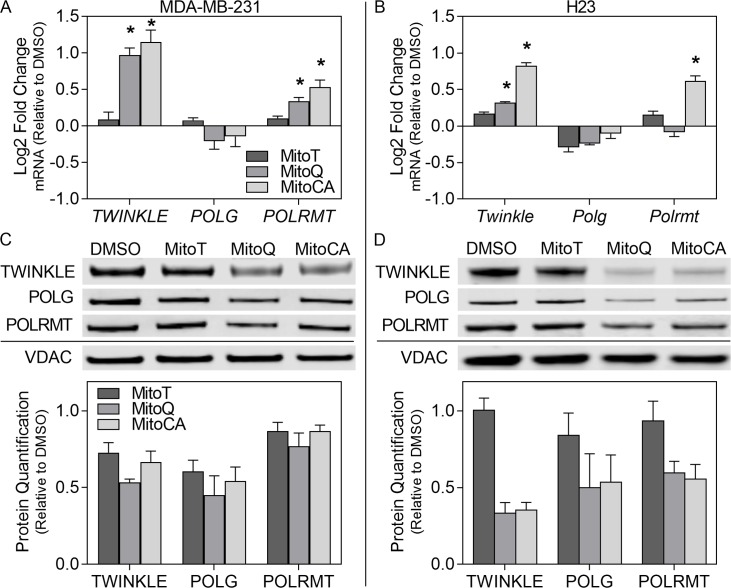
Mitochondrial replication machinery. Gene expression (A,C) and protein levels (B,D) for TWINKLE, POLG and POLRMT in MDA-MB-231 (A,C) and H23 (B,D) cell lines determined by qPCR and immunoblotting. Cells were exposed to DMSO (0.02%), MitoT (dark gray bars), MitoQ (gray bars), and MitoCA (light gray bars) at 2μM for 24 hours. In A-B, bars represent the average log2 fold change normalized to *GAPDH* and the DMSO control. Statistical significance is expressed as asterisks at p<0.05 relative to the DMSO control. In C-D, bars denote the mean densitometry (+/-1 SEM) of immunoblots of mitochondrial fractions normalized to VDAC and the corresponding DMSO treatment. For each assay, two independent experiments were performed (n = 3).

We investigated transcriptional changes in nuclear-encoded mitochondrial proteins essential to replication and transcription including the helicase *TWINKLE*, the DNA polymerase-γ-*POLG* and the RNA polymerase *POLRMT* in an attempt to further understand the influence of MTAs on mtDNA integrity. In MDA-MB-231 cells, treatment with MitoQ and MitoCA resulted in significant increases, >0.97 log2 fold change in expression of *TWINKLE* and >0.34 log2 fold change in expression in *POLRMT* (p<0.05) but no significant change in *POLG* expression (Fig 4A). MitoT had no effect on the abundance of any transcript evaluated. H23 cells treated with MitoQ and MitoCA for 24 hours displayed increases in *TWINKLE* mRNA levels ranging between a 0.32 and 0.83 log2 fold changes but no change in *POLG* expression (Fig 4B). Additionally, only treatment with MitoCA increased expression of *POLRMT* at 0.62 log2 fold change (p<0.05). In H23 cells, MitoT treatment also did not change transcript levels for any of the genes evaluated (Fig 4B).

Proteins levels of the same genes were quantified to further assess the effect of MTAs on mtDNA integrity. In MDA-MB-231 cells, treatment with all MTAs caused reductions in all proteins evaluated ranging from 27–47% reductions in TWINKLE, 40–55% decreases in POLG and 13–23% losses in POLRMT (Fig 4C). H23 cells exposed to MitoQ and MitoCA displayed moderate decreases in TWINKLE ranging from 65–67%, POLG from 47–50% and POLRMT from 40–44% reductions (Fig 4D). Treatment with MitoT had no effect on TWINKLE or POLG (<10% change) but caused a 16% reduction in POLRMT levels. Together these results demonstrate that transcription does not necessarily imply translation or translocation. It is clear that with MTA treatment proteins essential to mtDNA integrity are not being maintained at optimal levels within the mitochondria, which likely has severe consequences on mtDNA integrity and copy number.

### 3.7 Aconitase TCA Cycle Activity

**Fig 5:**
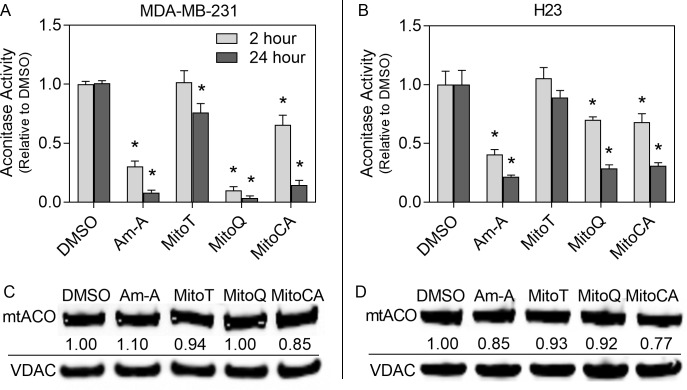
Mitochondrial TCA cycle aconitase activity. Aconitase activity was determined with the colorimetric aconitase enzyme activity assay in MDA-MB-231 (A) and H23 (B) cell lines exposed to either DMSO (0.02%), MitoT, MitoQ or MitoCA at 2μM or Antimycin A (Am-A) at 40μM for 2 (light) or 24 (dark) hours. Bars represent the relative mitochondrial aconitase activity normalized to mitochondrial protein and the DMSO control +/-1 SEM. Statistical significance is represented by asterisks with a p<0.05 relative to the DMSO control. In C-D, immunoblots of mitochondrial extracts were probed with anti-aconitase 2, and quantitated using densitometry and then normalized to VDAC and the DMSO treatment.

We measured mitochondrial TCA cycle activity via aconitase as an indicator of mitochondrial health. Recent studies have suggested that aconitase also has a role in mtDNA maintenance under oxidative stress through inactivation of its redox-sensitive iron-sulfur center [37–39]. The positive control Antimycin A significantly diminished aconitase activity at early and late time points in both cell types by 59–92% (p<0.05; Fig 5). Exposure of MDA-MB-231 cells to either MitoQ or MitoCA resulted in a rapid (<2 hours) and sustained decline (24 hours) in aconitase activity by 34–96% (p<0.05; Fig 5A). MitoT had no early impact on aconitase activity but did cause a 24% reduction after 24 hours exposure (p<0.05). In H23 cells, MitoQ or MitoCA treatment also resulted in a rapid (<2 hours) and sustained decline (24 hours) in aconitase activity by 30–71% (p<0.05; Fig 5B). MitoT had no effect on aconitase TCA cycle activity in H23 cells. While there was a substantial loss in mitochondrial TCA cycle activity, there was no corresponding loss in mitochondrial aconitase protein level (Fig 5C and 5D). Although there were minor decreases in aconitase protein in both cell types with MitoCA treatment (15–23%), these losses likely do not fully contribute to the decreased TCA cycle activity. All together, these results demonstrate enzymatic inhibition or a change of function of aconitase with MTA treatment from its known function in the TCA cycle and concurrently demonstrate decreased mitochondrial function.

### 3.8 Mitochondrial Respiration

**Fig 6:**
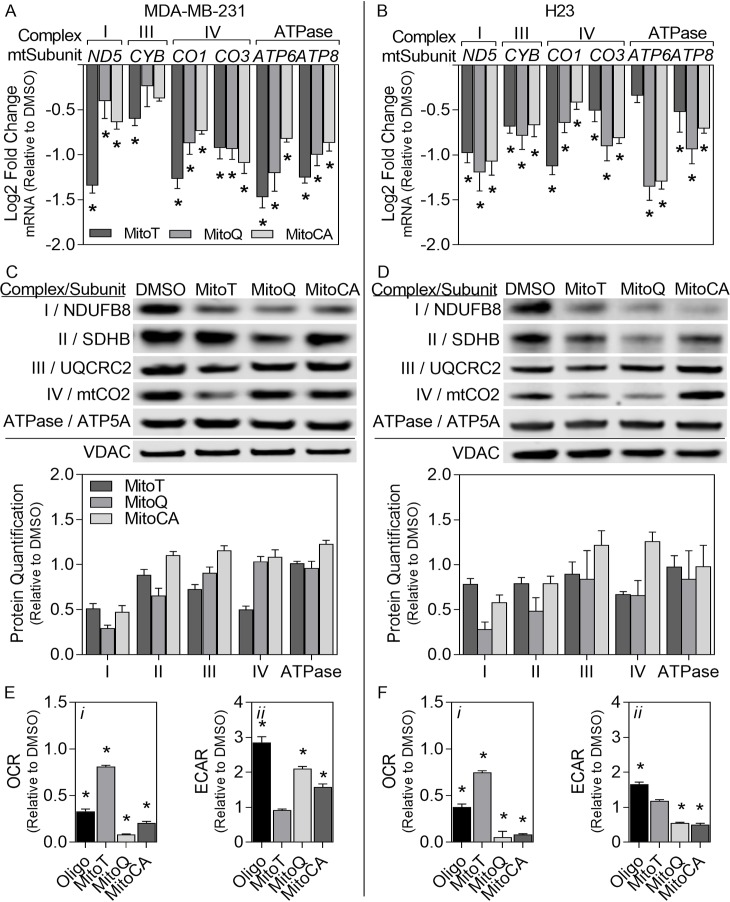
Mitochondrial respiration. The mRNA (A-B) and protein levels (C-D) levels for mitochondrial respiratory chain subunits were assessed using qPCR and immunoblotting. Mitochondrial bioenergetics were measured using a Seahorse XF^e^96 flux analyzer (E-F). MDA-MB-231 (A,C,E) and H23 (B,D,F) cells were exposed to either DMSO (0.02%), MitoT (dark gray bars), MitoQ (gray bars) or MitoCA (light gray bars) at 2μM for 24 hours. In C-D, mitochondrial complexes were probed with an antibody cocktail containing antibodies against all five mitochondrial complexes. Each band represents a different subunit of a mitochondrial complex. Band intensities were quantitated using densitometry. Bars denote the average mRNA log2 fold change normalized to *GAPDH* (A-B), the mean densitometry normalized to VDAC [(C-D) represented in Fig 4], the average oxygen consumption rate [OCR (E-F*i*)] or the average extracellular acidification rate [ECAR (E-F*ii*)] relative to the DMSO control +/-1 SEM. In A-B and E-F, statistical significance (p<0.05) with respect to the DMSO control is expressed with asterisks above each bar. For each assay, two independent experiments were performed (n = 3).

To determine the effects of MTA exposure on mitochondrial transcription, we measured the mRNA levels of subunits within RCCs encoded in mtDNA for both breast and lung cancer cell lines after 24 hours exposure (Fig 6A and 6B). There are no mitochondrial-encoded subunits of complex II. In MDA-MB-231 cells, all MTA treatments resulted in significant decreases in all complexes evaluated ranging from -0.40 to -1.5 log2 fold changes except for complex III (*CYB*) with MitoQ and MitoCA treatments. Similar changes were observed in H23 cells where all MTAs significantly down-regulated all complexes measured ranging from -0.42 to -1.4 log2 fold changes except for *ATP6* transcripts in MitoT treatments. All together, these data demonstrate that MTA treatment leads to a decrease in the mRNA levels of mitochondrial-encoded RCC subunits in both the breast and lung cancer cell types evaluated but that the effects on specific complexes are MTA and cell type specific.

To further confirm that MTA treatment results in the loss of RC subunits, we investigated changes in mitochondrial protein levels for RCCs (see fractionation efficiency in S5 Fig). In MDA-MB-231 cells treated with MitoQ, there was a decrease in subunits encoding complexes I (by 71%) and II (by 34%); the points at which electrons enter the RC. Additionally, with MitoCA, there was a 53% reduction in complex I and a minor increase in the ATPase at 23% but no change in other RC subunits evaluated. Interesting, treatment with MitoT caused a 49% reduction in complex I, a 27% loss in III and a 50% decrease in IV (Fig 6C). In H23 cells, MitoQ treatment triggered a decrease in complex I by 72%, in II by 52% and in complex IV by 34% (Fig 6D). MitoCA also caused a reduction in complexes I and II by 42% and 21%, respectively. However, MitoCA also resulted in a minor increase in complexes III by 22% and IV by 26%. Lastly, treatment with MitoT produced reductions in complexes I, II and IV by 21–33% (Fig 6D). These findings further demonstrate that MTAs have a downstream effect on RCC subunits which likely result in impaired OxPhos. Moreover, specific responses of each complex were dependent on the type of MTA used, though all MTAs significantly decreased complex I in both the breast and lung cancer cells examined.

### 3.9 Mitochondrial Bioenergetics

As MMP is a driving force for mitochondrial metabolism and dysregulation in OxPhos enhances excessive ROS production, we investigated MTA induced mitochondrial respiratory dysfunction via OCR and ECAR. The positive control Oligomycin, an ATPase inhibitor, caused a rapid decrease (>63%) in the OCR with a corresponding increase in the ECAR (>56%) after 1 hour exposure in both cell lines (p<0.05). After 24 hours exposure to MitoQ and MitoCA, MDA-MB-231 cells demonstrated greater than 79% reduction in OCR with a concurrent increase in ECAR by greater than 60% (p<0.05; Fig 6E). Although there was a decrease in OCR with MitoT by 19% (p<0.05) there was no corresponding increase in ECAR. In H23 cells treated with MitoQ or MitoCA, there was a greater than 92% decrease in OCR and also a decrease in ECAR by greater than 45% (p<0.05; Fig 6F). With MitoT treatment there was a 25% reduction in OCR (p<0.05) and a 19% non-significant increase in ECAR in H23 cells. The decreased mitochondrial oxygen consumption with all MTAs in both cell lines suggests mitochondrial dysfunction in the OxPhos chain. The impaired OxPhos coincided with increased ECAR in MDA-MB-231 cells suggesting a switch in energy production to glycolysis in this cell line. However, this response was not observed in H23 cells where ECAR either did not change or decreased with MTA treatment, suggesting a lower glycolytic capacity or enhanced MTA-induced stress in this cell line.

## 4. Discussion

The objective of this study was to evaluate the effect that TPP^+^ conjugated redox-active MTAs have on mitochondrial physiology and mtDNA integrity from a mechanistic chemotherapeutic perspective. Our results indicate that all MTAs substantially impact mtDNA integrity and mitochondrial homeostasis in both the breast and lung cancer cell lines evaluated. All tested TPP^+^ conjugated MTAs impacted mitochondrial physiology through alterations in superoxide levels or mitochondrial membrane depolarization, both of which are essential to proper mitochondrial function. In this study, treatment with MitoQ and MitoCA resulted in a rapid increase in superoxide levels (< 2 hr) and mitochondrial depolarization (< 1 hr). MitoT displayed varied responses between the two cell lines where MDA-MB-231 cells exhibited late mitochondrial membrane depolarization (24 hours) but no excess ROS production and the reverse was observed in H23 cells. The rapid surge in ROS with the concordant mitochondrial charge dissipation from these redox-active agents suggests that early responses may partially drive the downstream physiological responses observed on mtDNA integrity and mitochondrial homeostasis.

Treatment with all MTAs in this study resulted in significant increases in mtDNA degradation in both cell lines (Fig 2A and 2B). All MTAs also caused a decrease in mtDNA copy number when MMP was lost (3–8%) (Fig 1C and 1D & Fig 2C and 2D). Interestingly, MitoT treatment had no effect on ROS levels but still caused greater than 50% DNA fragmentation and a 3% reduction in copy number in MDA-MB-231 cells, possibly as a consequence of late (24 hour) mitochondrial depolarization. Additionally, MitoT treated H23 cells exhibited a minor, but early (4 hour) increase in ROS accompanied later (24 hours) by significant mtDNA fragmentation yet no loss in MMP or copy number (Fig 2B and 2D). Based on these findings we hypothesize that oxidant-induced mtDNA damage does not necessarily reduce mtDNA copy number, challenging, at least in cancer cells, the prevalent theory that enhanced oxidant induced damage leads to reductions in the highly redundant mtDNA genome [9] though this warrants further investigation. Together this data suggests that both charge separation and maintained redox homeostasis are essential for preserving mtDNA integrity. These data also stipulate that MMP is critical for conserving mtDNA copy number possibly due to the availability of charge-dependent nuclear-encoded mitochondrial proteins for maintaining mtDNA nucleoids.

MTAs induced changes in mtDNA gene expression (*7S*, *12S* and *16S*) perhaps by alterations in TFAM and SSBP1 protein levels, which in turn may contribute to mtDNA instability and decreased mitochondrial homeostasis. In this study, MTAs resulted in a decrease in TFAM (with the exception of MitoT treated MDA-MB-231 cells) and decreased ribosomal subunit (*12S* and *16S*) transcript levels (Fig 3) suggesting impaired transcription and decreased mtDNA nucleoid stability with TPP^+^ conjugated redox-active compounds. Furthermore, SSBP1 and 7S DNA are known to play an essential role in maintaining and synthesizing the D-loop for triple stranded mtDNA replication and transcription. At transcription initiation, 7S DNA is primed with *7S* RNA which is known to stably bind to the template DNA following transcription [40]. Additionally, cells depleted of SSBP1 are unable to generate 7S DNA and have been shown to have reduced mtDNA copy numbers [40]. In this investigation, SSBP1 protein and *7S* transcript levels were reduced in both cell lines as a consequence of MitoQ and MitoCA exposure (Fig 3), but either no change or only a slight increase was observed with MitoT. These results support that MitoQ, MitoCA and to a much lesser extent MitoT, impair mtDNA nucleoid and D-loop stability possibly through impaired transcription of mtDNA encoded transcripts and/or reduced import of nuclear encoded proteins like SSBP1 and TFAM due to charge dissipation.

The redox-active MTAs all had an impact on the levels of the nuclear-encoded mitochondrial proteins which make up the core replisome for mtDNA [41, 42]. Mitochondrial DNA-directed RNA polymerase (POLRMT) provides RNA primers for mtDNA replication and is the only RNA polymerase known to function in mitochondrial transcription [41–43]. The helicase TWINKLE has been directly associated with mtDNA copy number [36, 40] and D-loop formation through 7S DNA [40]. Mutations and knockdowns/outs in both DNA polymerase-γ (POLG) and TWINKLE have been observed with increased mtDNA damage and decreased mtDNA copy number both *in vitro* and *in vivo* [44–49]. The primary mechanism for targeting proteins to the mitochondria is through the addition of a cleavable mitochondria-targeted (pre)sequence (MTS) [13]. TWINKLE, POLG and POLRMT all contain a MTS [50–52]. This is significant as depolarization of the mitochondrial membrane can result in the inability to import proteins containing a MTS, which constitute approximately 70% of mitochondrial-directed proteins [13]. Mitochondrial depolarization thereby can inhibit turnover and repair mechanisms necessary for proper mitochondrial maintenance and function. Decreases in all MTS-containing proteins were observed with all MTAs except when MMP was maintained (in MitoT treated H23 cells) (Fig 4). Furthermore, the decrease in mitochondrial protein levels could also be due to decreased cytosolic translation, however this requires additional investigation. Collectively, this identifies a miscommunication between the nucleus and mitochondria that may be due to impaired protein translation or decreased import into mitochondria owing to the dissipation of the MMP, which likely have downstream severe consequences on mtDNA integrity and copy number.

Our results confirm that the MTAs studied had a negative impact on mitochondrial homeostasis as determined by changes in aconitase TCA cycle activity. Aconitase was used as an indicator for mitochondrial TCA cycle activity however aconitase is also known to couple metabolic regulation to mtDNA maintenance under oxidative stress where aconitase can bind to mtDNA in a similar manner to the high mobility group protein Abf2p; a functional homolog of TFAM in yeast [37]. There were significant decreases in mitochondrial aconitase activity when MMP was lost but this did not translate into significant decreases in protein levels (Fig 5), which could suggest complete inhibition or a change of function of aconitase.

Mitochondrial oxidative respiration was evaluated as MMP is the driving force for OxPhos, and dysfunctional OxPhos is known to produce excessive free oxygen radicals. All MTAs tested resulted in decreases in RCCs both at the mRNA and protein level (Fig 6A–6D) having variable effects dependent on the MTA and cell line. However, all MTAs consistently reduced complex I, the main entry point for protons and electrons OxPhos. Since decreased RCC protein and mRNA levels do not imply reduced functionality, we investigated changes in OCR and ECAR. Impaired OxPhos was confirmed by severely decreased OCRs with MitoQ and MitoCA (by >79% reduction) and moderate reductions in OCRs with MitoT (by >19% reduction; Fig 6E and 6F) in both cell lines. In MDA-MB-231 cells when OxPhos was severely impaired, ECAR was significantly enhanced suggesting a switch in energy production to glycolysis in this cell line (Fig 6E). The same observation was not observed in H23 cells (Fig 6F). It is well accepted that cancer cells generate ATP dominantly via aerobic glycolysis, known as the “Warburg effect” [53, 54] so it would not be surprising if redox-active presumed antioxidants, functioning as pro-oxidants, enhance this process indirectly by impairing OxPhos. Additionally, more aggressive carcinomas (illustrated by the MDA-MB-231 cell line) are known to be more dependent on glycolysis than OxPhos for ATP production [55] which could partially explain the differential responses we observed between the two cell lines examined here. It is probable that less aggressive carcinomas like H23 cells have lower glycolytic capacities and are therefore unable to compensate metabolically when OxPhos is impaired. It is also possible that TPP^+^ conjugated MTAs effect H23 cells to a greater degree since both OxPhos and glycolytic endpoints decreased with treatment (except with MitoT where the effects were milder; Fig 6F).

We propose two potential mechanisms that could act in concert or independently to induce mitochondrial dysregulation and degradation in response to TPP^+^ conjugated MTAs. We hypothesize that the primary drivers are the excess generation of superoxide and/or mitochondrial membrane depolarization. In the ROS-mediated mechanism, excess superoxide results in damaged mitochondrial proteins and nucleic acids, which can ultimately result in impaired mtDNA replication, transcription, translation and RC function, leading to mitochondrial dysregulation. In the MMP-mediated mechanism, charge dissipation can result in the decreased import of newly synthesized nuclear-encoded mitochondrial-directed proteins containing a charge dependent MTS. This in turn can result in reduced mitochondrial protein levels and reduced turnover of damaged proteins, which could enhance mitochondrial dysfunction similar to the ROS mechanism. When the two mechanisms interplay, we expect the effects to be more dramatic (as observed with MitoQ and MitoCA treatments), the reduction of mitochondrial proteins with the simultaneous increase in ROS could increase mitochondrial protein and mtDNA damage and degradation, and at the same time, decrease mtDNA copy number and mitochondrial transcription and translation. As a consequence, MTAs could induce OxPhos dysregulation and decreased mitochondrial homeostasis and potentially lead to mitochondrial loss.

## 5. Conclusions

In this study we demonstrated that the mitochondria-targeted redox-sensitive therapeutics mitoTEMPOL, mitoquinone and mitochromanol-acetate impair mitochondrial homeostasis through decreased mtDNA integrity and oxidative respiration in triple negative breast (MDA-MB-231) and small cell lung (H23) cancer cell lines. These MTAs likely exploit anti-cancer activity through mechanisms involving mitochondrial membrane depolarization and/or surges in mitochondrial superoxide levels. Although these compounds were initially identified as antioxidants [2], this study has shown that they can function as pro-oxidants to varying degrees; therefore further studies are needed to understand the long term impact of these compounds on cancer cell mitochondrial physiology and overall cellular health to understand their therapeutic potential alone or in combination with established chemotherapeutic agents.

## Supporting Information

S1 Fig Anticancer activity of MitoQ. (A) Anticancer activity profile for MitoQ across 60 cancer cell lines as determined by the sulforhodamine B (SRB) assay in the NCI-60 panel. Z-scores for each cell line are relative to the average GI50 over all cell lines (Z-score = 0). Bars to the right indicate drug sensitivity while bars to the left indicate drug resistance. (B) Dose response curves for MitoQ in select lung (including H23) and breast cancer (including MDA-MB-231) cell lines. Cell growth was evaluated by the SRB assay after 72 hours with increasing concentrations of MitoQ.
(TIF)

S2 Fig Anticancer activity of MitoCA. (A) Anticancer activity for MitoCA across the NCI-60 cancer cell line panel as measured by the SRB assay. Z-scores are relative to the average GI50 over all cell lines (Z-score = 0). Bars to the right and left indicate drug sensitivity and resistance, respectively. (B) Dose response curves for MitoCA in select lung (including H23) and breast cancer (including MDA-MB-231) cell lines. Cell growth was evaluated by the SRB assay after 72 hours exposure to increasing concentrations of MitoCA.
(TIF)

S3 Fig Baseline mitochondrial superoxide production. MDA-MB-231 (dark) and H23 (light) cell lines were assayed for superoxide using the fluorometric MitoSOX assay after seeding at the same cell density. Bars represent the average normalized fluorescence of at least two independent experiments (n = 3). Statistical significance is indicated as ** p<0.01.
(TIF)

S4 Fig Baseline MMP microscopy. DIC and pre-treatment TMRM imaging in MDA-MB-231 cells for each condition in Fig 1E. TMRM imaging was used to acquire baseline images for evaluating changes in MMP in MDA-MB-231 cells after 1 hour drug exposure (as in Fig 1C and 1D; see Fig 1E for 1 hour time point). Scale bar is 20μm.
(TIF)

S5 Fig Mitochondrial and cytoplasmic fractionation efficiency. Immunoblots were prepared in MDA-MB-231 (A) and H23 (B) cells to evaluate the extraction efficiency of crude mitochondrial and cytosolic protein extractions. Mitochondrial and cytosolic protein was extracted after 24 hours with 2μM MitoT, MitoQ or MitoCA or 0.02% DMSO and analyzed via SDS-PAGE. Immunoblots were probed with antibodies against VDAC (mitochondrial) and α-Tubulin (cytosolic) as loading controls for determination of contamination.
(TIF)

S1 Table TaqMan Assays, Antibodies and Primer Sequences. This table contains the primers sequences that were used for PCR, and antibodies used for immunoblot.
(DOC)
