# Supplementary figures and images for: Therapeutic Targeting of the Mitochondria Initiates Excessive Superoxide Production and Mitochondrial Depolarization Causing Decreased mtDNA Integrity
Source: PLoS One. 2016 Dec 28;11(12):e0168283. doi: 10.1371/journal.pone.0168283 (PMC5193408; doi:10.1371/journal.pone.0168283)

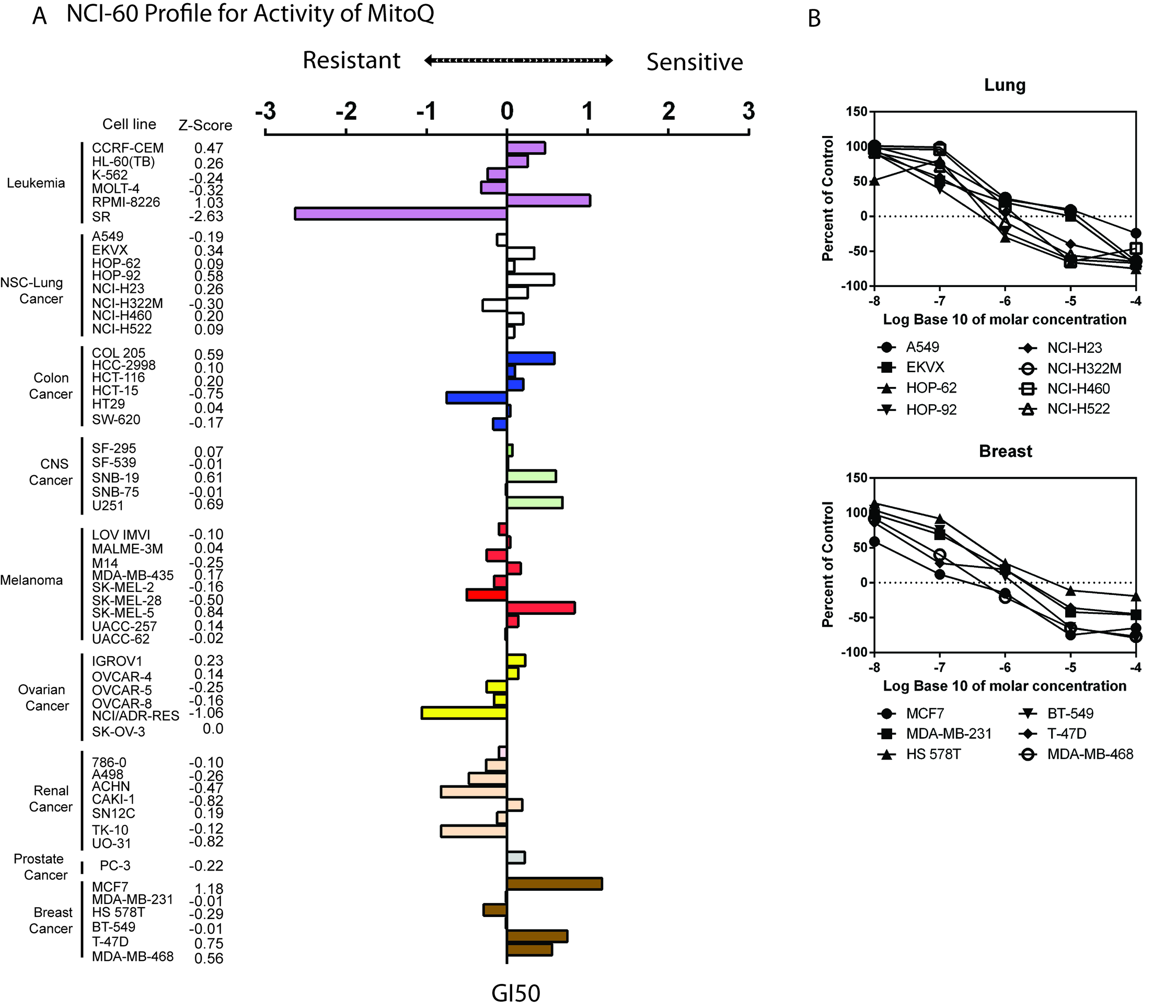

Supplement: S1 Fig — (A) Anticancer activity profile for MitoQ across 60 cancer cell lines as determined by the sulforhodamine B (SRB) assay in the NCI-60 panel. Z-scores for each cell line are relative to the average GI50 over all cell lines (Z-score = 0). Bars to the right indicate drug sensitivity while bars to the left indicate drug resistance. (B) Dose response curves for MitoQ in select lung (including H23) and breast cancer (including MDA-MB-231) cell lines. Cell growth was evaluated by the SRB assay after 72 hours with increasing concentrations of MitoQ. (TIF) [file pone.0168283.s001.tif]

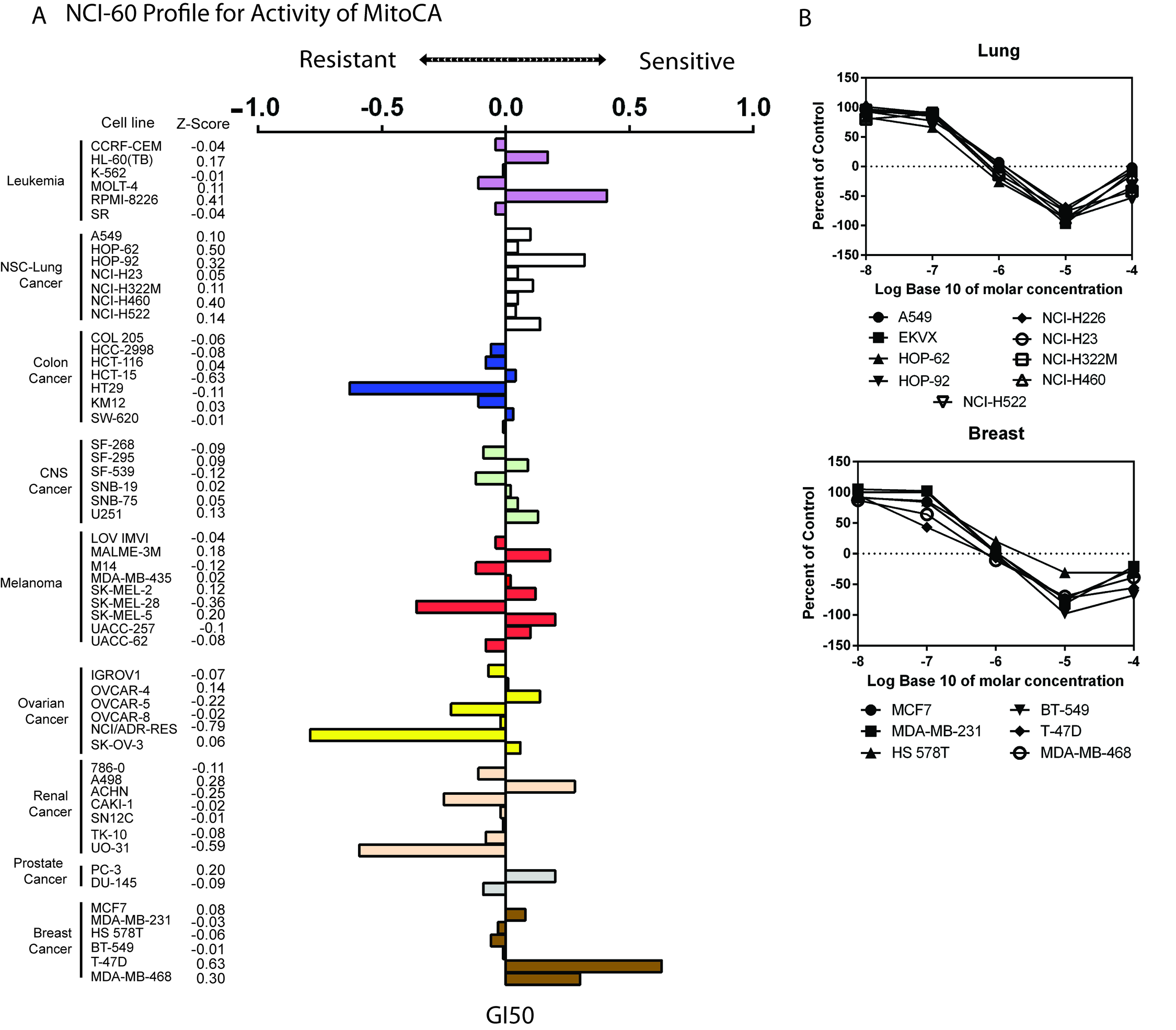

Supplement: S2 Fig — (A) Anticancer activity for MitoCA across the NCI-60 cancer cell line panel as measured by the SRB assay. Z-scores are relative to the average GI50 over all cell lines (Z-score = 0). Bars to the right and left indicate drug sensitivity and resistance, respectively. (B) Dose response curves for MitoCA in select lung (including H23) and breast cancer (including MDA-MB-231) cell lines. Cell growth was evaluated by the SRB assay after 72 hours exposure to increasing concentrations of MitoCA. (TIF) [file pone.0168283.s002.tif]
