# Supplementary material for: Therapeutic Targeting of the Mitochondria Initiates Excessive Superoxide Production and Mitochondrial Depolarization Causing Decreased mtDNA Integrity
Source: PLoS One. 2016 Dec 28;11(12):e0168283. doi: 10.1371/journal.pone.0168283 (PMC5193408; doi:10.1371/journal.pone.0168283)
Supplement: S1 Table — This table contains the primers sequences that were used for PCR, and antibodies used for immunoblot. (DOC) [file pone.0168283.s006.doc]

| **Gene** | **Company** | **TaqMan Assay ID** |
| --- | --- | --- |
| *mt-ND5* | Thermo Fisher | Hs02596878_g1 |
| *mt-CYB* | Thermo Fisher | Hs02596867_s1 |
| *mt-CO1* | Thermo Fisher | Hs02596864_g1 |
| *mt-CO3* | Thermo Fisher | Hs02596866_g1 |
| *mt-ATP6* | Thermo Fisher | Hs02596862_g1 |
| *mt-ATP8* | Thermo Fisher | Hs02596863_g1 |
| *mt-7S* | Thermo Fisher | Hs02596861_s1 |
| *mt-RNR1 (12S)* | Thermo Fisher | Hs02596859_g1 |
| *mt-RNR2 (16S)* | Thermo Fisher | Hs02596860_s1 |
| *TWINKLE (C10orf2)* | Thermo Fisher | Hs00958168_g1 |
| *POLG* | Thermo Fisher | Hs00160298_m1 |
| *POLRMT* | Thermo Fisher | Hs04187596_g1 |
| *GAPDH* | Thermo Fisher | Hs03929097_g1 |
| **Antibody** | **Company** | **Catalog Number** |
| OXPHOS Antibody Cocktail | Abcam | Ab110411 |
| ACONITASE [EPR8283(B)] | Abcam | Ab129105 |
| VDAC | Cell Signaling | 4661S |
| SSBP1 | EMD Millipore | ABN403 |
| TFAM | EMD Millipore | ABE483 |
| β-ACTIN 8H10D10 | Cell Signaling | 3700S |
| TWINKLE clone 13A10.1 | EMD Millipore | MABN814 |
| POLG [EPR7296] | Abcam | Ab128899 |
| POLRMT | Abcam | Ab32988 |
| α-TUBULIN | Cell Signaling | 2144S |
| **Target** | **Primer Sequence** | |
| Long mitochondrial region  (10kb product) | 5’-TCT AAG CCT CCT TAT TCG AGC CGA-3’  5’-TTT CAT CAT GCG GAG ATG TTG GAT GG-3’ | |
| Short mitochondrial region  (117bp product) | 5’-AAG TCA CCC TAG CCA TCA TTC TAC-3’  5’-GCA GGA GTA ATC AGA GGT GTT CTT-3’ | |
| *β-2-Microglobulin*  (107bp product) | 5’-TGC TGT CTC CAT GTT TGA TGT ATC T-3’  5’-TCT CTG CTC CCC ACC TCT AAG T-3’ | |
| *tRNA_LEU_*  (86bp product) | 5’-CAC CCA AGA ACA GGG TTT GT-3’  5’-TGG CCA TGG GTA TGT TGT TA-3’ | |
